# Decoding osteosarcoma pathogenesis: the pivotal influence of m^6^A modification

**DOI:** 10.1080/15592294.2026.2705608

**Published:** 2026-07-21

**Authors:** Chaomei Huang, Zhongyu Han, Junyan Su, Meiqi Zhang, Yumeng Lin, Haibei He, Yu Tang, Peng Guo, Yi Wang, Haoran Chen

**Affiliations:** a Department of Rehabilitation Medicine, Chengdu Xinhua Hospital Affiliated to North Sichuan Medical College, Chengdu, China; b Institute of Nephrology, Zhongda Hospital, Southeast University, Nanjing, China; c Department of Rehabilitation Medicine, The First People’s Hospital of Longquanyi District, Chengdu, China; d Center of Gerontology and Geriatrics, West China Hospital, Sichuan University, Chengdu, China; e Department of Nanjing Tongren Eye Center, Nanjing Tongren Hospital, School of Medicine, Southeast University, Nanjing, China

**Keywords:** Osteosarcoma, m^6^A, metabolic reprogramming, programmed cell death, tumor microenvironment

## Abstract

Osteosarcoma (OS) is the predominant primary malignant bone tumor in children and adolescents. Current treatments mainly include surgical resection combined with chemotherapy, but they still cannot effectively control tumor metastasis and recurrence, and may pose a risk of long-term complications. N6-methyladenosine (m^6^A) is the most widespread internal modification. It orchestrates various biological and disease-related events via the reversible m^6^A machinery comprising writers, erasers and readers. As a key epigenetic regulatory mechanism, m^6^A modification opens new avenues for deeper exploration of OS mechanisms and for developing new therapeutic targets and biomarkers. In this manuscript, we systematically explore the critical roles of m^6^A in OS cell proliferation, metastasis, metabolic reprogramming, programmed cell death, and tumor microenvironment regulation. Our aim is to provide a robust theoretical framework to underpin future basic studies and clinical applications in this field.

## Introduction

Cancer remains a significant worldwide health issue that threatens human health. Despite notable advancements in medicine and technology that have improved survival rates for many cancers, some types still have low survival rates, falling short of desired outcomes. Osteosarcoma (OS) is the primary malignant bone tumor and one of the cancers with the lowest survival rates among children and adolescents. Epidemiological data reveals that in American, the incidence rate of OS is 4.1/million among children aged 0–14 and 7.9/million among adolescents aged 15–19, with a 5-year survival rate of roughly 65% [1]. At present, the standard therapeutic approach consists of a combination of surgical resection and chemotherapy. However, due to the aggressive and metastatic nature of OS, approximately 20% of patients experience metastasis and recurrence [2]. Additionally, since OS predominantly affects children and adolescents, the treatment tends to increase the risk of early and severe chronic health conditions among long-term survivors [2, 3]. For instance, a longitudinal study revealed that childhood OS survivors face a significantly elevated risk of cardiovascular damage compared to the control group, with 18% of survivors experiencing major cardiovascular damage before the age of 50 [2]. Therefore, there is an urgent need to develop new therapeutic approaches to enhance the prognosis for OS patients.

Epigenetic modification is a genetic mechanism that influences gene expression and function through chemical changes to DNA or its associated proteins (such as histones), all without altering the DNA sequence [4]. These modifications, including DNA methylation, non-coding RNA (ncRNA) regulation, and histone modification, greatly add to the intricacy of gene regulation and are vital in numerous physiological and pathological processes, including heredity, development, and disease [5].

Epigenetic alterations are a notable feature of cancer. By modulating gene expression without changing the DNA sequence, they give cancer cells the ability to adjust to the tumor microenvironment (TME), elude immune surveillance, and cultivate drug resistance [6, 7]. This makes them a major driver of cancer progression, and the reversibility of these changes offers new treatment approaches. For instance, using targeted therapies to eliminate epigenetic abnormalities in cancer cells and restore normal gene expression patterns can hold back tumor growth and metastasis [8]. Among epigenetic modifications, N6-methyladenosine (m^6^A) has gained attention for its role in various biological processes, including cancer [9]. M^6^A is the most common form of post-transcriptional modification in eukaryotic mRNA and is crucial for gene expression and RNA metabolism regulation. By interacting with various ‘writers,’ ‘erasers,’ and ‘readers,’ m^6^A dynamically modulates mRNA stability, splicing, and translation efficiency, thereby affecting protein synthesis and participating in various processes. In cancer, m^6^A modification is capable of interfering with the biological activities of various genes, influencing tumor cell proliferation, metastasis, programmed cell death (PCD) and other malignant behaviors, and has become a critical factor in cancer [9].

While the function of m^6^A in driving OS progression is well-documented, its precise mechanisms remain to be thoroughly explained. In this manuscript, we delve into the latest research advancements to clarify how m^6^A modulates tumor growth, metastasis, metabolic reprogramming, PCD, and the TME in OS.

## M^6^ A modification

M^6^A is a highly dynamic and reversible modification that acts on the N6 position of adenine (A) in RNA molecules [10]. Its sites are mainly distributed in the 3’UTR and translation stop codon regions, typically conforming to the 5’-RRACH-3’ motif (*R* = G/A, H = A/C/U), whereas m^6^A modification in the 5’UTR region is relatively rare [11]. M^6^A exerts broad regulatory effects on various RNA types, including mRNA and ncRNA (tRNA, lncRNA, miRNA, circRNA), and is crucial for key RNA metabolic steps [12]. In m^6^A modification, the dynamic deposition, removal, and functional interpretation of the methyl mark are orchestrated by three protein classes: writer methyltransferases, eraser demethylases, and reader m^6^A-binding proteins [13].

The writer complex, with core members methyltransferase like 3 (METTL3), METTL14, and Wilms’ tumor 1-associated protein (WTAP), catalyzes the addition of m^6^A modifications to RNA [14]. Other related proteins like zinc finger CCCH-type containing 13 (ZC3H13), KIAA1429, and RNA binding motif protein 15/15B (RBM15/15B) also participate in this process by aiding enzyme complex positioning or substrate recognition, determining the specific positions and degree of methylation [15–17].

METTL3 is a key ‘writer’ in m^6^A methylation, playing a crucial and irreplaceable role. Bokar et al. initially isolated the m^6^A methyltransferase complex (MTC) in 1994; the complex has three components and requires all of them to be present to be active [14]. Subsequently, in 1997, they identified METTL3 as the key catalytic subunit within the MTC [18]. Full-length METTL3 has 580 amino acids and two key domains: zinc-finger domain (ZFD) and methyltransferase domain (MTD) [19]. The ZFD comprises two CCCH-type zinc-finger structures, each with three conserved cysteines and one histidine. It can specifically recognize RNA regions rich in 5’-GGACU-3,’ promoting METTL3 recruitment to specific RNA sequences [20]. The MTD has a classic Rossmann fold, a common structural trait in methyltransferases, and contains S-adenosyl methionine/S-adenosyl homocysteine (SAM/SAH)-binding pockets [21]. The SAM-binding pocket specifically attaches to SAM, using it as a methyl donor to catalyze m^6^A [22]. SAH is produced when SAM donates a methyl group in the methylation reaction. The SAH-binding pocket can bind SAH molecules, promptly removing SAH to sustain the high efficiency of m^6^A [23]. However, METTL3 alone exhibits low catalytic activity and needs to combine with METTL14 and WTAP to facilitate the full functionality of MTC. METTL14 does not possess a SAM-binding site and exhibits no catalytic activity [24]. Instead, it critically stabilizes METTL3’s conformation. Within the MTC, METTL14 and METTL3 form a stable 1:1 heterodimer [25]. This interaction enhances substrate RNA binding and boosts catalytic activity. WTAP recruits the heterodimer to specific nuclear locations, promoting its binding to target RNA [26]. In addition, the cofactors KIAA1429 and RBM15/15B are also involved in recruiting the MTC to specific locations on target RNA [17, 27].

Erasers, which include fat mass and obesity-associated protein (FTO), α-ketoglutarate-dependent dioxygenase homolog 5 (ALKBH5), and ALKBH3, mediate the removal of m^6^A from RNA, enabling reversible RNA methylation regulation [28–30]. The interplay between writers and erasers allows for dynamic control of m^6^A modification.

**Figure 1.**
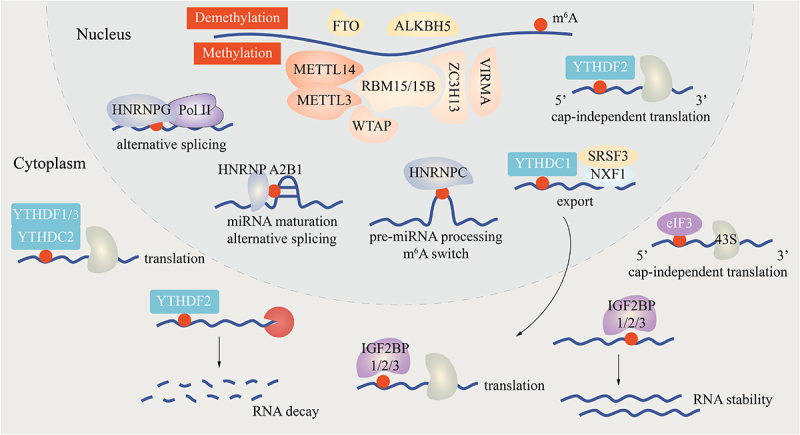
Introduction of m^6^A RNA modification complex. the m^6^A RNA modification system centers around a methyltransferase complex whose core components include METTL3, METTL14, and WTAP. Proteins such as RBM15, RBM15B, ZC3H13, and VIRMA may also be associated with this complex. The removal of m6A modifications is mediated by demethylases, including FTO and ALKBH5. Reader proteins that recognize m^6^A modifications comprise YTHDF1/2/3, YTHDC1/2, IGF2BP1/2/3, and HNRNPC/A2B1. m^6^A modification is involved in regulating multiple RNA-related processes, such as miRNA maturation alternative splicing, pre-miRNA processing m6A switch, RNA decay, RNA translation and RNA stability.

Readers specifically recognize m^6^A-modified RNA and regulate different processes through their binding effects [13]. Key m^6^A-binding proteins include YT521-B homology (YTH) domain-containing family proteins and insulin-like growth factor 2 mRNA binding proteins (IGF2BPs). Different readers exert distinct effects after recognizing m^6^A. For example, YTHDF1 enhances translation, YTHDF2 promotes degradation, and YTHDF3 synergistically amplifies the effects of YTHDF1/2 [31]. YTHDC1 facilitates RNA splicing and nuclear export, whereas YTHDC2 enhances RNA translation efficiency [32, 33]. Additionally, IGF2BPs contribute to RNA stabilization (Figure 1) [34].

## The role of m^6^A in OS

Based on the critical function of m^6^A in gene regulatory processes, its dysregulation can broadly impact gene expression and drive OS progression via various mechanisms. Studies have revealed that aberrant m^6^A participates in regulating OS-related processes like cell proliferation, invasion, metastasis, angiogenesis, PCD, and TME modulation.

### M^6^A and proliferation of OS

The autonomous acquisition of sustained proliferative signaling is one of the most fundamental differences between tumor and normal cells. Normal cells enter G1/S phase only periodically and proliferate in an orderly manner under strict external control: they require exogenous growth factors, cell–cell contact signals, and the precise restraint of negative-feedback loops [35, 36]. Tumor cells bypass these constraints and achieve ligand-independent, self-sustained proliferation through three converging strategies: autocrine synthesis and secretion of growth-promoting ligands, ligand-independent receptor activation that directly triggers proliferative pathways, and active suppression of negative-feedback circuits to release proliferative brakes [37–39]. M^6^A-mediated epigenetic changes are critical for tumor proliferation. By targeting the mRNAs associated with oncogenes and cell cycle regulatory factors, they reshape the proliferative properties of tumor cells at multiple levels and drive the abnormal proliferation of tumor cells.

Zhang et al. reported that METTL3 exhibits high expression in OS cell lines compared to normal osteoblasts. METTL3 facilitates the m^6^A modification of the lncRNA metastasis associated lung adenocarcinoma transcript 1 (MALAT1), thereby enhancing MALAT1 stability and subsequently augmenting MALAT1-driven OS cell proliferation. Knockout of MALAT1 can inhibit the effects caused by METTL3 overexpression [40]. Zhang et al. reported that METTL3 overexpression promotes the methylation and stability of constitutive photomorphogenic-9 signalosome subunit 5 (COPS5) mRNA, leading to increased proliferation of OS cells [41]. Similarly, An et al. reported that overexpressing or knocking down METTL3 causes a respective increase or decrease in the protein levels of zinc finger and BTB domain containing 7C (ZBTB7C), thereby modulating the proliferative capacity of OS cells [42]. COPS5 overexpression correlates with the suppression of multiple tumor suppressor factors like p53, p57, and p27, and is closely linked to adverse prognoses in various cancers [43–45]. Liang et al. reported that METTL3 overexpression boosts m^6^A methylation of lymphoid enhancer binding factor 1 (LEF1) mRNA, activates the Wnt/β-catenin pathway, and promotes OS proliferation [46]. Kim et al. found that METTL3 knockdown can lead to altered splicing in about 1800 genes, particularly those linked to the cell cycle, and impacts OS proliferation and cycle progression through splicing factors like SFPQ [47]. GDNF receptor α1 (GFRA1) exhibits elevated expression levels in various cancers, promoting cell proliferation and survival via AMPK, PI3K-AKT, and other pathways [48–50]. Kim et al. reported that WTAP promotes circ_0032463 generation and stability through m^6^A modification. Circ_0032463 competitively relieves the suppressive effect of miR-145-5p on GFRA1, thereby driving OS progression [51]. RBM15 can also mediate the m^6^A methylation of lncRNA THAP9-AS1, thereby promoting the malignant progression of OS [52].

**Figure 2.**
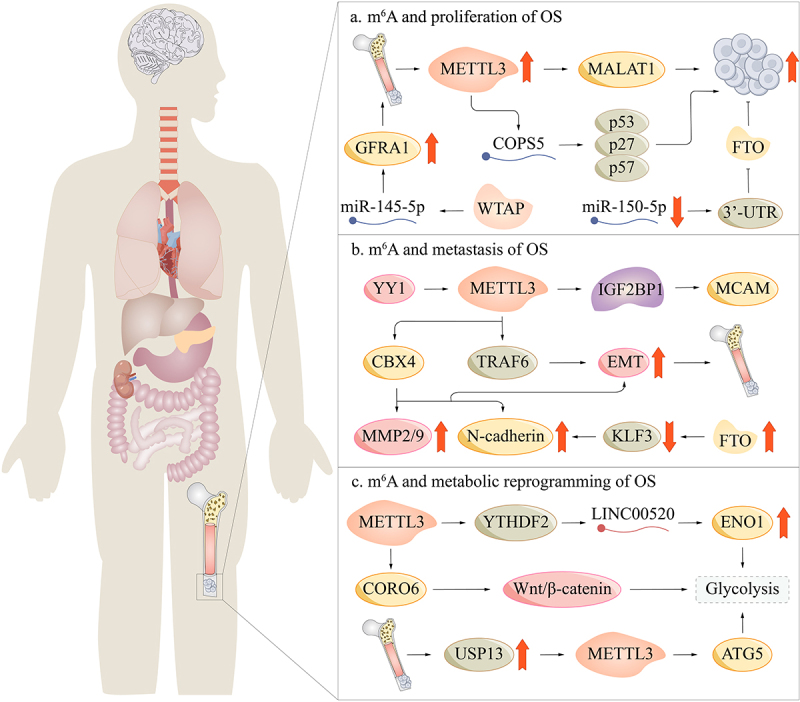
The role of m^6^A modification in osteosarcoma pathogenesis. ATG5: autophagy gene autophagy related 5; CBX4: chromobox homolog 4; COPS5: constitutive photomorphogenic-9 signalosome subunit 5; CORO6: coronin 6; EMT: epithelial-mesenchymal transition; ENO1: α-enolase; FTO: fat mass and obesity-associated protein; IGF2BP1: insulin-like growth factor 2 mRNA binding protein 1; KLF3: Krüppel like factor 3; MALAT1: metastasis associated lung adenocarcinoma transcript 1; METTL3: methyltransferase like 3; MMP2/9: matrix metalloproteinase 2/9; GFRA1: GDNF receptor α1; TRAF6: TNF receptor-associated factor 6; USP13: ubiquitin-specific protease 13; WTAP: Wilms’ tumor 1-associated protein; YTHDF2: YT521-B homology domain family member 1; YY1: yin yang 1.

Similarly, m^6^A erasers and readers can also influence OS cell proliferation. Xu et al. reported that FTO exhibits elevated expression in OS and facilitates OS cell proliferation, whereas FTO knockdown leads to the opposite outcome. In addition, miR-150-5p exhibits reduced expression in OS cells. Targeting the 3′-UTR of FTO enables the repression of FTO expression, thereby blocking the proliferation of OS cells [53, 54]. Shan et al. reported that FTO suppresses Krüppel like factor 3 (KLF3) expression by destabilizing KLF3 mRNA through demethylation, and promotes OS cell proliferation by upregulating cyclin D1 and downregulating p21 [55]. Yang et al. reported that reduced ALKBH5 expression is significantly linked to adverse prognosis in OS patients. YTHDF2 specifically binds to m^6^A-modified suppressor of cytokine signalling-3 (SOCS3) mRNA, promoting its degradation. Overexpressing ALKBH5 lowers the methylation on SOCS3 mRNA, reducing its degradation and thereby increasing SOCS3 protein levels [56]. This sequence of events suppresses the signal transducer and activator of transcription 3 (STAT3) pathway, causing inhibited OS proliferation [57]. However, Chen et al. demonstrated that ALKBH5 overexpression can also block YTHDF2-mediated degradation of the oncogenic lncRNA plasmacytoma variant translocation 1 (PVT1). This process further elevates PVT1 expression levels, ultimately promoting OS proliferation via multiple pathways [58, 59]. Yadav et al. reported that ALKBH5-mediated demethylation elevates the expression of histone deubiquitinase ubiquitin specific peptidase 22 and ubiquitin ligase RING finger protein 40. This in turn suppresses H2A monoubiquitination and activates oncogenes, leading to unregulated cell cycle, persistent replication, and DNA repair in OS (Figure 2(a)) [60].

### M^6^A and metastasis of OS

As a signature trait of malignant tumors, metastasis is the main reason for cancer-related deaths. During tumor metastasis, epithelial-mesenchymal transition (EMT) is crucial for helping tumor cells gain migratory and invasive capabilities: Normal epithelial cells maintain tight intercellular adhesion through E-cadherin, while tumor cells specifically reduce E-cadherin expression and increase the expression of mesenchymal markers like N-cadherin and vimentin [61]. This enables them to transform into a mesenchymal phenotype with weak adhesiveness and strong migratory ability–a transition that allows them to more easily break through the basement membrane and extracellular matrix (ECM), thereby initiating the invasion stage [62]. Additionally, ECM degradation (which opens up migration channels for tumor cells) and neovascularization (which provides pathways for tumor cell dissemination) are also indispensable key links in tumor metastasis, collectively driving the completion of the metastatic process [63].

M^6^A modification is an important regulator of OS metastasis. MCAM, also known as CD146, is overexpressed in various malignancies like OS, colorectal cancer, and breast cancer, etc. By mediating intercellular adhesion, regulating angiogenesis, and modulating the TME, it notably elevates the ability of tumor cells to metastasize [64–66]. Du et al. reported that under the regulation of the transcription factor yin yang 1 (YY1), METTL3 strengthened methylation of MCAM mRNA and facilitated the expression of MCAM. The YY1-METTL3-IGF2BP1-MCAM axis is pivotal for driving OS progression, particularly in facilitating invasion and metastasis [64]. Wang et al. reported that METTL3 expression is even higher in OS cells with greater metastatic activity. METTL3 facilitates the expression of TNF receptor-associated factor 6 (TRAF6), which in turn enhances the invasive ability, metastatic potential, and EMT process of OS cells [67]. Inhibition of METTL3 reduces vimentin expression and increases E-cadherin expression. Huo et al. reported that METTL3 mediates the m^6^A modification of chromobox homolog 4 (CBX4), thereby enhancing its stability. CBX4 increases matrix metalloproteinase 2/9 (MMP2/9) and N-cadherin while decreasing E-cadherin, thus activating the EMT pathway, and thereby promoting OS metastasis [68]. Shan et al. reported that FTO overexpression boosts N-cadherin and reduces E-cadherin by destabilizing KLF3 mRNA, thereby promoting the EMT process in OS [55]. The overexpression of KLF3 can reverse these changes. Cao et al. found that miR-451a is reduced in OS cells. Overexpression of miR-451a suppresses YTHDC1, influencing 3-phosphoinositide dependent protein kinase 1 (PDPK1) mRNA m^6^A and inhibiting OS progression. Mechanistically, YTHDC1 stabilizes PDPK1 mRNA, and PDPK1 activates the AKT-mammalian target of rapamycin (mTOR) pathway by phosphorylating AKT at Thr308, thereby promoting OS proliferation, migration, and EMT [69]. Wei et al. reported that YTHDF1 overexpression is associated with worse TNM stage, distant metastasis, and lymphatic metastasis in OS. Bioinformatics analysis shows that YTHDF1 can recognize the methylation sites of CCR4-NOT transcription complex subunit 7 (CNOT7) and promote its expression [70]. The METTL3-CNOT7-YTHDF1 axis affects the deadenylation process of mRNA, thereby regulating mRNA degradation and influencing the metastasis of OS cells (Figure 2(b)) [71].

### M^6^A and metabolic reprogramming of OS

Metabolic reprogramming represents the metabolic adaptation undergone by tumor cells to facilitate rapid proliferation. Key characteristics of this reprogramming include aberrant glucose metabolism, enhanced glutamine metabolism, and accelerated lipid and nucleotide metabolism [72].

As a defining feature of abnormal glucose metabolism in tumors, the Warburg effect is particularly notable. This effect is reflected in tumor cells’ tendency to utilize glycolysis as an energy-generating pathway, even under oxygen-rich conditions, which diminishes their dependence on the more efficient mitochondrial oxidative phosphorylation pathway [73]. Despite its lower efficiency in ATP production, glycolysis offers a swift energy supply that sustains tumor cell proliferation [74]. Furthermore, glycolysis intermediates function as essential precursors for biomacromolecules, and these precursors are crucial for tumor cell proliferation and their adaptation to the TME [75].

M^6^A is critically involved in the aerobic glycolysis of OS. Liu et al. revealed that METTL3-YTHDF1 axis stabilizes and elevates coronin 6 (CORO6) expression in OS, consequently activating the Wnt/β-catenin axis and strengthening glycolytic activity to facilitate malignant progression [76]. Wei et al. reported that METTL3 enhances the stability of LINC00520 RNA, thereby sustaining its high expression. LINC00520 directly binds to the glycolytic key enzyme α-enolase (ENO1), blocks its ubiquitination, and increases ENO1 protein stability [77]. Upregulation of ENO1 in turn activates aerobic glycolysis and metabolic reprogramming. Wang et al. found that ubiquitin-specific protease 13 (USP13) and METTL3 exhibit high expression levels in OS tissues, with both being associated with poor clinical outcomes. Immunoprecipitation and mass spectrometry analysis showed that USP13 eliminates K48-linked ubiquitin chains at the K488 site of METTL3, thereby enhancing the stability of the METTL3 protein. METTL3 binds to and stabilizes the key autophagy gene autophagy related 5 (ATG5) mRNA through m^6^A modification. The USP13/METTL3/ATG5 pathway promotes autophagy, thereby enhancing cellular metabolic flexibility, survival, and glycolytic reprogramming [78]. The USP13 inhibitor Spautin-1 induces METTL3 degradation, significantly inhibiting OS growth and metastasis. Mei et al. showed that YTHDC1 interacts with specific m^6^A sites on the mRNAs of phosphofructokinase and lactate dehydrogenase A (two key glycolytic enzymes), which boosts the stability of these mRNAs and drives glycolysis. N-acetyltransferase 10 (NAT10) is overexpressed in OS and further promotes glycolysis by enhancing YTHDC1 mRNA stability and translation [79]. Similarly, Liu et al. reported that YTHDF3 is overexpressed in OS and binds to 3-phosphoglycerate kinase 1 (PGK1) mRNA, enhancing its stability and consequently accelerating the glycolysis of OS cells [80]. Yang et al. reported that circCTNNB1 is highly expressed in OS and interacts with RBM15. Through m^6^A modification, this interaction promotes the expression of hexokinase 2 (HK2), glucose-6-phosphate isomerase, and PGK1, which in turn facilitates the glycolysis and drives the malignant progression and lung metastasis of OS [81]. Similarly, circARHGAP12, a novel m^6^A-modified circRNA, exhibits high expression in OS. It can bind to c-Myc mRNA, enhancing its stability and promoting glycolysis and doxorubicin resistance (Figure 2(c)) [82].

Cancer cells need increased lipid quantities to maintain their fast proliferation rate, as lipids are essential for cell membrane expansion, organelle biogenesis, and the production of signaling molecules. This elevated demand drives hyperactivity of the de novo lipogenesis pathway [83]. In cancer cells, key enzymes involved in this pathway, namely acetyl-CoA carboxylase (ACC) and fatty acid synthase (FASN), are generally upregulated [84–86]. While research in OS is lacking, inhibiting de novo lipogenesis via m^6^A modification shows promise in curbing tumor growth. Studies in hepatocellular carcinoma reveal that FTO knockout boosts FASN’s m^6^A level, prompting YTHDF2 to degrade FASN mRNA [87, 88]. This cascade suppresses lipogenesis, inhibits tumor cell growth, and triggers apoptosis. Conversely, FTO activation improves m^6^A demethylation, causing lipid accumulation [89]. Another study reported that m^6^A reader HNRNPA2B1 promotes the growth and proliferation of esophageal squamous cell carcinoma by up-regulating the expression of fatty acid synthases ATP citrate lyase (ACLY) and ACC1 [90].

Enhanced glutamine metabolism is a hallmark of abnormal amino acid metabolism in tumor cells [91]. Tumor cells convert glutamine to glutamate through glutaminase, which is subsequently transformed into α-ketoglutarate (α-KG) to enter the tricarboxylic acid (TCA) cycle, thereby supplying the cell with energy and precursors for biosynthesis [92]. To sustain a functional TCA cycle, tumor cells typically upregulate glutaminase. In clear cell renal cell carcinoma, the loss of the tumor suppressor gene von Hippel–Lindau (VHL) is a hallmark [93]. Xiao et al. discovered that VHL and FTO exhibit a synthetic lethal relationship [94]. VHL inactivation causes VEGF and PDGF to be constitutively activated, and these factors further target the downstream glutamine transporter SLC1A5 to promote glutamine metabolism [95]; SLC1A5 is a key target of FTO. Knockdown of FTO increases m^6^A methylation of SLC1A5 mRNA, thereby reducing SLC1A5 expression and inhibiting the growth of VHL-deficient renal cancer cells. However, investigations into m^6^A regulation in OS remain scarce.

### M^6^A and PCDs of OS

PCD is an active and ordered cell death mechanism in multicellular organisms, which encompasses diverse types like apoptosis, pyroptosis, ferroptosis, autophagy, etc [96]. Tumor cells usually secure survival advantages through resisting PCDs; therefore, restoring or enhancing the sensitivity of tumor cells to PCDs has become an important strategy for tumor therapy.

Apoptosis is the first discovered form of PCD, and it is defined by features like cell shrinkage, nuclear fragmentation, and apoptotic body formation [97]. The apoptotic process is primarily triggered via the mitochondrial and death receptor pathways, involving a cascade reaction of a series of caspases. Apoptotic resistance is a key mechanism that helps tumor cells gain survival advantages and develop resistance to radiotherapy and chemotherapy [98].

m^6^A modification can influence the apoptotic pathways in OS cells via multiple signaling pathways. Zhou et al. reported that METTL3 knockdown significantly decreases the anti-apoptotic protein Bcl-2, while elevating the pro-apoptotic protein Bax and the apoptotic executor caspase-3, thereby promoting OS cell apoptosis [99]. Liu et al. reported that METTL14 is reduced in OS. Elevated METTL14 activates caspase-3, thereby significantly promoting OS cell apoptosis [100]. Notably, these effects can be reversed by caspase-3 inhibitor Z-VAD-FMK. In contrast, Yuan et al. reported that ALKBH5 is diminished in OS. ALKBH5 promotes OS apoptosis via its m^6^A demethylase activity; specifically, it reduces the methylation of pre-miR-181b-1 and Yes-associated protein 1 (YAP1) transcripts, ultimately lowering YAP protein levels [101]. As the key effector of the Hippo pathway and an important oncogene in numerous cancers, YAP1 is a major driver of chemoresistance in OS cells and is crucial for suppressing apoptosis [102–104]. Yang et al. reported that ALKBH5 overexpression reduces SOCS3 mRNA methylation, thereby impairing its recognition and degradation by YTHDF2 and increasing SOCS3 mRNA stability and protein abundance. Elevated SOCS3 markedly inhibits STAT3 phosphorylation, leading to reduced expression of the STAT3 downstream Bcl-2 and cell-cycle arrest at the G0–G1 phase. Knocking down YTHDF2 produces a similar effect, and ALKBH5 shows no additional impact in YTHDF2-silenced cells [56]. Lv et al. reported that FTO is overexpressed in OS and boosts the demethylation of dishevelled binding antagonist of β-catenin 1 (DACT1) mRNA, thereby reducing DACT1 expression [105]. This activates the Wnt/β-catenin axis, facilitating proliferation and suppressing apoptosis in OS cells [106].

Ferroptosis is an iron-dependent non-apoptotic PCD form, characterized by the abnormal buildup of lipid peroxidation products and distinct changes in mitochondrial morphology [107]. The core trigger of this process is the impairment of the cellular antioxidant defense system; on this basis, intracellular iron overload induces the production of massive reactive oxygen species (ROS) via the Fenton reaction. These ROS then drive the continuous accumulation of lipid peroxides, causing cell death [108].

The ferroptosis process in OS cells can be influenced by m^6^A modification, which achieves this by modulating the expression of ferroptosis-related genes. Huang et al. reported that methionine adenosyltransferase 2 A (MAT2A) and RBM15 are highly expressed in OS. RBM15 induces methylation of MAT2A, thereby promoting OS metastasis and inhibiting ferroptosis [109]. Knocking down MAT2A or RBM15 in OS cells suppresses GPX4 expression, reduces GSH levels, and enhances the accumulation of Fe^2+^ and ROS, thereby spurring ferroptosis. Similarly, Xie et al. reported that KIAA1429 directly regulates the 3′UTR of nuclear factor erythroid 2–related factor 2 (Nrf2) through m^6^A modification [110]. Silencing KIAA1429 inhibits the viability and invasion of OS cells and promotes sulfasalazine-induced ferroptosis by inhibiting the Nrf2/NAD(P)H:quinone oxidoreductase 1 (NQO1) axis. Chen et al. reported that METTL5-YTHDF1-mediated m^6^A methylation stabilizes ubiquitin protein ligase E3C (UBE3C) mRNA, increasing UBE3C levels and promoting AHNAK ubiquitination and degradation. Reducing UBE3C levels hinders OS cell proliferation and accelerates ferroptosis, with these impacts being counteracted by Fer-1 [111].

The biological function of autophagy in tumors is not uniform but exhibits a ‘double-edged’ property. The final effect of autophagy depends on the tumor’s developmental phase, the dynamic alterations of the TME, and the self-regulatory activity level of autophagy [112]. On one hand, it inhibits tumor progression by removing damaged organelles and misfolded proteins within cells to prevent the accumulation of harmful substances that trigger DNA mutations; meanwhile, it maintains genomic stability, blocks abnormal cell proliferation, and can initiate the ‘autophagic cell death’ program in abnormal cells [113]. On the other hand, in the hypoxic and nutrient-deficient TME, autophagy can degrade its own components, thereby supplying energy and raw materials to support the survival and proliferation processes of tumor cells. During chemotherapy and radiotherapy, it can also reduce the killing effect and lead to tumor drug resistance; it may even facilitate tumor metastasis through metabolic reprogramming and influencing the TME [114].

Multiple m^6^A-related regulatory axes have been identified to mediate autophagy and regulate OS malignant progression. As mentioned, the USP13/METTL3/ATG5 axis stabilizes the autophagy-related gene ATG5 by inhibiting the decay of ATG5 mRNA, thereby promoting autophagy and the malignant OS progression [78]. Additionally, in OS, the expression of miR-451a is downregulated. This downregulation activates the AKT/mTOR pathway through the YTHDC1/PDPK1 axis, thereby promoting OS cell proliferation and EMT [69]. Notably, mTOR is a key negative regulator in the regulation of autophagy: activated mTOR can directly phosphorylate the autophagy-related protein ATG13, which reduces the binding ability between UNC-51 like autophagy activating kinase 1 (ULK1) and ATG13 [115, 116]. This further causes the inhibition of ULK1 kinase activity and ultimately suppresses the initiation of autophagy. In another study, Liu et al. reported that tripartite motif containing 17 (TRIM17) is significantly upregulated in OS, and it promotes the ubiquitination and degradation of FTO protein and enhances the m6A modification of pyruvate dehydrogenase kinase 1 (PDK1) mRNA, thereby activating the AKT/mTOR signaling pathway and driving the malignancy of OS [117]. Furthermore, Zhang et al. reported that the METTL3-YTHDF1 axis participates in the regulation of DNA-dependent protein kinase catalytic subunit (DNA-PKcs) expression, enhancing the resistance of OS cells to anlotinib. Mechanistically, anlotinib treatment can upregulate METTL3, subsequently promotes the m^6^A methylation of PRKDC mRNA; the modified PRKDC mRNA then binds to YTHDF1 to facilitate the expression of DNA-PKcs. Subsequently, DNA-PKcs mediates protective autophagy in OS cells and their resistance to anlotinib by interacting with Beclin-1 and modulating its ubiquitination [118].

**Figure 3.**
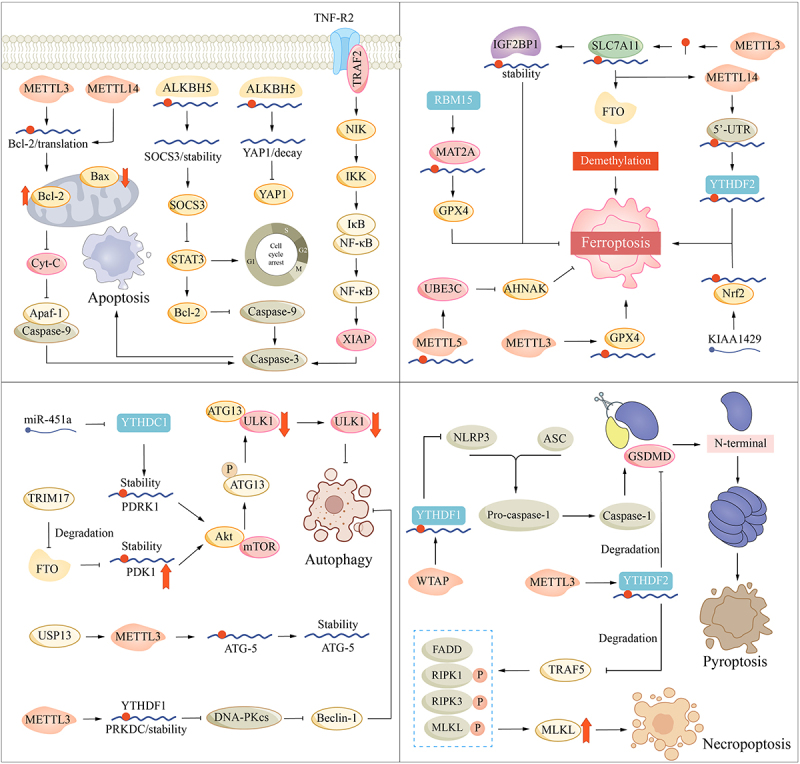
The mechanism of m^6^A regulating cell apoptosis, ferroptosis, autophagy, pyroptosis and necroptosis.

Beyond the aforementioned PCDs, m^6^A modification may further influence OS progression through the regulation of other PCD types such as pyroptosis and necroptosis; however, current research in this field remains relatively scarce. Nevertheless, existing relevant research findings in other types of tumors may provide potential directions for investigations in the field of OS. For instance, WTAP-mediated m^6^A modification of NOD-like receptor pyrin domain-containing 3 (NLRP3) inhibits the NLRP3/caspase-1/Gasdermin-D (GSDMD) axis, thereby suppressing pyroptosis and exacerbating colorectal cancer progression [119]; the METTL3/YTHDF2 axis can directly promote the degradation of GSDMD mRNA, inhibit pyroptosis, and facilitate the malignant progression of breast cancer [120]; while FTO reduces the m^6^A modification level of NLRP3 mRNA, maintains the stability of NLRP3 mRNA, promotes its protein expression, and further activates NLRP3/Caspase-1/GSDMD-dependent pyroptosis, thereby overcoming cisplatin resistance in ovarian cancer [121]. In addition, the increased expression of METTL3 mediated by M2 phenotype tumor-associated macrophages (TAMs) promotes the m^6^A-dependent degradation of TRAF5 mRNA, inhibits TRAF5-mediated necroptosis, and thereby enhances the oxaliplatin resistance of colorectal cancer cells [122]. Collectively, these findings from other tumor types underscore the potential regulatory role of m^6^A in pyroptosis and necroptosis, offering valuable references to guide future in-depth investigations into its mechanisms in OS progression (Figure 3).

Akt : protein kinase B; Apaf-1 : apoptotic peptidase activating factor 1; ASC : apoptosis-associated speck-like protein containing a CARD; ATG-5/13 : Autophagy Related 5/13; Bcl-2 : B-cell lymphoma 2; Bax : BCL2-associated X protein; Cyt-C : cytochrome C; DNA-PKcs : DNA-dependent protein kinase catalytic subunit; FADD : Fas-associated death domain protein; FTO : fat mass and obesity-associated protein; GSDMD : gasdermin D; GPX4 : glutathione peroxidase 4; IGF2BP1 : insulin-like growth factor 2 mRNA binding protein 1; mTOR : mammalian target of rapamycin; METTL3/5/14 : methyltransferase like 3/5/14; MLKL : mixed lineage kinase domain-like protein; NLRP3 : NOD-like receptor pyrin domain-containing 3; Nr2 : nuclear factor erythroid 2-related factor 2; PDK1 : pyruvate dehydrogenase kinase 1; RBM15 : RNA binding motif protein 15; RIPK1/3 : receptor interacting serine/threonine kinase 1/3; SLC7A11 : solute carrier family 7 member 11; STAT3 : signal transducer and activator of transcription 3; TRAF2/5 : TNF Receptor Associated Factor 2/5; TRIM17 : tripartite motif containing 17; ULK1 : UNC-51 like autophagy activating kinase 1; USP13 : ubiquitin-specific protease 13; UBE3C : ubiquitin protein ligase E3C; WTAP : Wilms tumor 1-associated protein; YTHDC1 : YTH domain containing 1; YTHDF1/2 : YTH domain family member 1/2; ZBTB32 : zinc finger and BTB domain containing 32.

### M^6^A and TME of OS

The TME refers to the multicellular environment surrounding tumor cells, which comprises stromal cells, immune cells, signal transduction molecules, ECM, and blood vessels [123]. The TME is deeply involved in tumor progression through multiple mechanisms: it supplies tumor cells with physical support and essential nutrients; it stimulates tumor proliferation and enhances tumor invasion and metastasis capabilities by secreting signaling molecules; and it helps tumors resist chemotherapy, targeted therapy, and immunotherapy by constructing physical barriers and inducing an immunosuppressive state [124]. The TME of OS exhibits significant immunosuppressive characteristics, containing various immunosuppressive cell subtypes, such as TAMs, cancer-associated fibroblasts (CAFs), myeloid-derived suppressor cells (MDSCs), regulatory T cells, and exhausted T cells [125]. M^6^A can directly regulate the malignant traits of tumor cells, and it can also indirectly modulate tumor onset and progression by affecting diverse cellular components and molecular networks in the TME.

TAMs are among the key immune cell types that infiltrate the TME, and come in two subtypes: M1 and M2 [126]. Among them, the M2 subtype plays a crucial pro-tumor role, including promoting tumor proliferation, facilitating angiogenesis and ECM formation, and mediating immune suppression [127]. Liang et al. reported that METTL3 is overexpressed in OS. It stabilizes regulators of G-protein signaling 1 (RGS1) mRNA, which promotes OS cell invasion and proliferation, suppresses apoptosis, and drives macrophages toward an M2 phenotype with elevated transforming growth factor-β (TGF-β) and IL-10 [128]. Silencing METTL3 can suppress the M2 polarization effect, and overexpressing RGS1 is capable of reversing this suppressive effect.

CAFs are a key component in the TME, promoting tumor growth, invasion, and metastasis through various mechanisms [129]. For example, CAFs secrete angiogenesis factors like VEGF to improve tumor blood vessel formation, release enzymes such as MMPs to break down the matrix and facilitate tumor metastasis, and produce immunosuppressive factors to shield tumor cells from immune attack [130–132]. Cancer cells induce the conversion of mesenchymal stem cells (MSCs) to CAFs by secreting TGF-β1 [131, 133]. METTL3 stabilizes TGF-β1 mRNA via m^6^A modification, promoting its expression and driving this differentiation [133].

M^6^A-associated lncRNAs also serve as epigenetic regulators influencing the development and functional maintenance of immune cells. DCGR5 has been recognized as a lncRNA closely associated with OS prognosis, exhibiting a strong correlation in expression levels with ZBTB32 [134]. ZBTB32, a key transcription factor and tumor suppressor gene, is often underexpressed in various tumors, a condition linked to cancer development and unfavorable prognosis [134]. Functionally, ZBTB32 significantly influences the differentiation and functional stability of diverse immune cells. During the maturation of B cells into antibody-secreting plasma cells, ZBTB32 directly binds to the *CIITA* gene (MHC-II transcription activator), inhibiting its expression and the activity of downstream MHC class II genes, thereby precisely controlling plasma cell differentiation [135]. In T cells, ZBTB32 modulates proliferation and differentiation, restrains excessive immune responses, and influences T cell exhaustion [136, 137]. In NK cells, ZBTB32 antagonizes the inhibitory factor B lymphocyte-induced maturation protein 1 (Blimp-1), thus regulating NK cell proliferation [138]. Additionally, Bi et al. reported that several types of m^6^A-associated LncRNA are closely related to the OS TME. Bioinformatics analysis revealed correlations between lncRNAs and immune cell infiltration: TNS1-AS1/TFPI2-DT with positive correlations to memory/naive B cell infiltration; LINC01474 (positive) and LINC00910 (negative) with CD8^+^ T cell infiltration; and LINC00538 with positive correlation to resting dendritic cell (DC cell) infiltration and negative correlation to activated DC cell infiltration [139]. Another bioinformatics analysis established a risk prediction model containing 6 lncRNAs [140]. Among these lncRNAs, decreased levels of lncRNA AC004812.2 significantly correlated with unfavorable overall survival in OS patients. Pearson analysis demonstrated that AC004812.2 expression were positively associated with both IGF2BP1 and YTHDF1. It is thus hypothesized that AC004812.2 is likely to contribute to OS progression by modulating m^6^A modification. In addition, the low-risk group exhibited markedly elevated monocyte infiltration relative to the high-risk group, whereas the abundance of other immune subsets–including macrophages, T cells and NK cells–showed no significant difference between the two groups.

**Figure 4.**
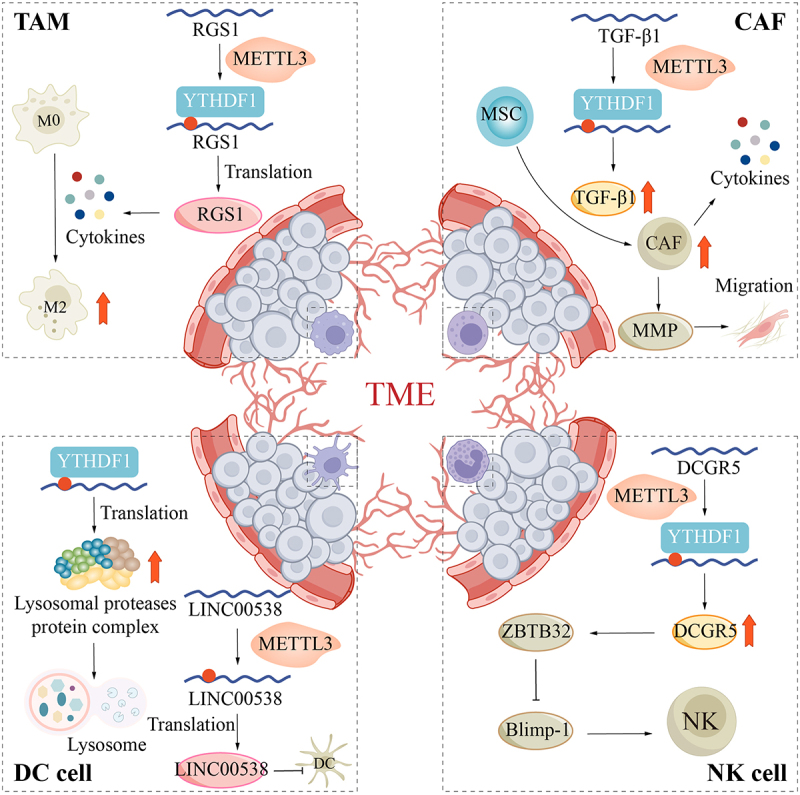
The role of m^6^A modification in tumor microenvironment and immune cells. blimp-1 : B lymphocyte induced maturation protein 1; CAF : cancer-associated fibroblast; DC cell : dendritic cell; METTL3 : methyltransferase like 3; MMP : matrix metalloproteinase; MSC : mesenchymal stem cell; NK cell : natural killer cell; RGS1 : regulator of G-protein signaling 1; TAM : tumor-associated macrophage; TGF-β1 : transforming growth factor-β1; TME: tumor microenvironment; YTHDF1: YTH domain family member 1; ZBTB32: zinc finger and BTB domain containing 32.

Fan et al. found that both RAS-like proto-oncogene A (RALA) mRNA and protein levels were markedly higher in OS tissues and derived cell lines than in normal bone tissue or osteoblasts. High expression of RALA alters the immune infiltration status in the OS TME, increasing the infiltration of neutrophils, macrophages, and Th2 cells while decreasing the infiltration of DC cells, NK cells, and Th17 cells [141]. Meanwhile, RALA expression is highly correlated with the degree of m^6^A methylation. Alterations in m^6^A-related gene expression may lead to the abnormal high expression of RALA. Methylation sites are present in both the upstream and downstream regions relative to the RALA transcription start site, and their methylation levels are negatively correlated with RALA expression [141]. These findings indicate that m^6^A can affect immune infiltration in OS by regulating RALA. However, its exact molecular mechanism remains incompletely understood and requires more in-depth research (Figure 4, Table 1).

**Table 1.** The function of m6A methylation molecules in OS.

Process	Molecule	Role	Target RNA	Mechanism	References
Proliferation	METTL3	Oncogene	MALAT1	Enhance MALAT1 stability and promote MALAT1-driven cell proliferation	[40]
		Oncogene	COPS5	Increase COPS5 stability and inhibit multiple tumor suppressors such as p53, p57, and p27	[41]
		Oncogene	ZBTB7C	Enhance ZBTB7C stability and promote ZBTB7C-driven cell proliferation	[42]
		Oncogene	LEF1	Enhance LEF1 stability and activate the Wnt/β-catenin pathway	[46]
	WTAP	Oncogene	circ_0032463	Promote the stability of circ_0032463 and competitively abrogate the inhibitory effect of miR-145-5p on GFRA1	[51]
	RBM15	Oncogene	THAP9-AS1	Enhance THAP9-AS1 stability and promote THAP9-AS1-driven cell proliferation	[52]
	FTO	Oncogene	KLF3	Destabilize KLF3, leading to the upregulation of cyclin D1 and downregulation of p21	[55]
	ALKBH5	Suppressor	SOCS3	Reduce SOCS3 degradation and inhibit the STAT3 pathway	[56]
		Oncogene	PVT1	Reduce PVT1 degradation and promote PVT1-driven cell proliferation	[58,59]
		Oncogene	USP22, RNF40	Upregulate the expression of USP22 and RNF40, suppress H2A monoubiquitination, and activate oncogenes	[60]
Metastasis	METTL3	Oncogene	MCAM	Upregulate MCAM expression and regulate angiogenesis and cell-cell adhesion	[64]
		Oncogene	TRAF6	Upregulate TRAF6 expression and enhance cell invasive ability	[67]
		Oncogene	CBX4	Enhance CBX4 stability and increase the expression of MMP2/9 and N-cadherin	[68]
	YTHDC1	Oncogene	PDPK1	Increase PDPK1 stability and promote the AKT-mTOR pathway by phosphorylating AKT at Thr308	[69]
	YTHDF1	Oncogene	CNOT7	Upregulate CNOT7 expression and promote CNOT7-driven metastasis	[70]
Metabolic reprogramming	METTL3	Oncogene	CORO6	Upregulate CORO6 expression, activate the Wnt/β-catenin axis, and enhance glycolysis	[76]
		Oncogene	LINC00520	Enhance LINC00520 stability, inhibit ENO1 ubiquitination, and activate aerobic glycolysis	[77]
		Oncogene	ATG5	Increase ATG5 stability, promote autophagy, thereby enhancing cell survival and glycolytic reprogramming	[78]
	YTHDC1	Oncogene	LDHA, PFKM	Improve the stability of glycolytic key enzymes	[79]
	YTHDF3	Oncogene	PGK1	Enhance PGK1 stability and promote glycolysis	[80]
	RBM15	Oncogene	circCTNNB1	Upregulate the expression of HK2, glucose-6-phosphate isomerase, and PGK1	[81]
Apoptosis	METTL3	Oncogene	Bcl-2	Upregulate Bcl-2 expression and inhibit caspase-3	[99]
	METTL14	Suppressor	Caspase-3	Activate caspase-3 and promote cell apoptosis	[100]
	ALKBH5	Suppressor	YAP1	Inhibit YAP expression and suppress the Hippo pathway	[101]
		Suppressor	SOCS3	Reduce SOCS3 degradation, inhibit the STAT3 pathway and Bcl-2 expression	[56]
Ferroptosis	RBM15	Oncogene	MAT2A	Enhance MAT2A stability, upregulate GPX4 expression, and inhibit ferroptosis	[109]
	KIAA1429	Oncogene	Nrf2	Directly regulate the 3’UTR of Nrf2 and promote the Nrf2-NQO1 axis	[110]
	METTL5	Oncogene	UBE3C	Increased UBE3C stability, promoted AHNAK ubiquitination, and inhibited ferroptosis	[111]
Autophagy	METTL3	Oncogene	ATG5	Inhibit ATG5 degradation and promote protective autophagy	[78]
	YTHDC1	Oncogene	PDPK1	Activate the AKT-mTOR pathway, phosphorylate ATG13, leading to ULK1 inhibition and autophagy suppression	[69]
	FTO	Suppressor	PDK1	Inhibit PDK1, suppress the AKT-mTOR pathway, and promote autophagy	[117]
	YTHDF1	Oncogene	PRKDC	Enhance PRKDC stability, upregulate DNA-PKcs expression, regulate Beclin-1 ubiquitination, and mediate protective autophagy	[118]
TME	METTL3	Oncogene	RGS1	Enhance RGS1 stability, increase TGF-β and IL-10 levels, and drive macrophage M2 polarization	[128]
		Oncogene	TGF-β1	Enhance TGF-β1 stability and induce the differentiation of MSCs into CAFs	[133]
	DCGR5	Suppressor	ZBTB32	Upregulate the expression of ZBTB32 and regulate the proliferation and differentiation of T cells, B cells, and NK cells	[134]

## Conclusion

OS continues to pose a significant challenge in clinical practice due to its high metastatic potential, poor prognosis, and long-term treatment-related complications. Current standard therapies fail to address the root causes of OS progression, highlighting a pressing demand for innovative treatments targeting essential molecular pathways driving OS malignancy. As a significant post-transcriptional epigenetic modification, m^6^A has become an essential influencer of OS pathophysiology, providing novel perspectives on OS pathogenesis and therapeutic development.

In this manuscript, we highlight m^6^A modification, a core mechanism in the field of RNA epigenetic regulation, and systematically explore its key roles and molecular mechanisms in the occurrence, development, and therapeutic response of OS. As the most abundant methylation modification type, the regulatory process of m6A relies on the synergistic action of three core protein complexes: writers, erasers, and readers. These three complexes jointly form a dynamic regulatory network, which is widely involved in multiple key biological processes in OS. In the aspect of cell proliferation, m6A can affect the progression of the cell cycle by regulating target genes (such as the Cyclin family and MYC). During the metastasis process, it can modulate the migratory capacity of OS cells by modifying EMT-related genes (such as PDRK1 and CBX4). In terms of metabolic reprogramming, m^6^A can target key genes in glucose metabolism (like PGK1 and HK2) and also regulate core genes in lipid metabolism (like ACC and FASN). Through reshaping glucose and lipid metabolism, it supports the high energy requirements for the rapid proliferation of OS cells. Meanwhile, m^6^A is also involved in regulating PCDs like apoptosis, ferroptosis, and autophagy by modulating key genes such as BCL-2, BAX (apoptosis), GPX4 (ferroptosis), and ATG5 (autophagy). Furthermore, by impacting the functions of diverse immune cells and the release of cytokines, it further worsens the development of an immunosuppressive TME in OS.

Based on the currently published data, m^6^A modification does not exhibit a single oncogenic or tumor-suppressive role in the progression of OS; instead, it shows significant dual functional characteristics. The core determinant of this functional bias lies in the biological properties of the downstream target genes modified by m^6^A: when m^6^A modification acts on oncogenes, it can enhance the stability of target mRNAs or promote their translation efficiency, thereby increasing the expression of oncogenes and ultimately driving the progression of OS. In contrast, when m^6^A modification targets tumor suppressor genes, it may accelerate the degradation of target mRNAs or hinder their translational process, lowering the expression levels of these genes and thus also driving tumor development. Only in certain specific scenarios can m^6^A exert a tumor-suppressive effect by regulating processes such as the nuclear translocation of tumor suppressor genes.

Despite significant progress, several gaps remain in our understanding of m^6^A in OS. First, the mechanisms by which m^6^A modulates OS lipogenesis and glutamine metabolism are largely uncharacterized. Lipogenesis supplies essential lipids required for membrane biosynthesis and signal transduction in OS cells undergoing rapid division, while glutamine metabolism supplies carbon and nitrogen sources for nucleotide synthesis and redox balance. However, it remains unknown which m^6^A ‘writers,’ ‘erasers,’ or ‘readers’ are involved in regulating key enzymes of these metabolic pathways (such as ACLY in lipogenesis or GLUD1 in glutamine metabolism). Further investigation, including transcriptomic and metabolomic analyses of OS cells with modulated m^6^A regulators, is required to fully map m^6^A-regulated metabolic networks and identify potential metabolic vulnerabilities in OS.

Second, although relevant studies in other cancer fields have demonstrated that m^6^A can regulate pyroptosis and necroptosis, it is currently unknown whether m^6^A modifies transcripts of key pyroptosis-related genes (such as NLRP3 and GSDME) or necroptosis-related genes (such as RIPK1 and MLKL) in OS, or if it indirectly regulates these pathways through upstream signaling molecules. Clarifying this mechanism could provide new strategies to augment the effectiveness of immunotherapy or chemotherapy in OS.

Third, current m^6^A research in OS primarily focuses on individual m^6^A components or linear regulatory axes, while the crosstalk between different m^6^A regulators requires systematic exploration. For instance, the synergy between ‘writers’ and ‘readers’ – such as whether the m^6^A methylation of a transcript mediated by METTL3 enhances its recognition by YTHDF1 to promote translation – and the antagonism between ‘writers’ and ‘erasers’ – such as whether FTO reverses METTL3-induced m^6^A modification of a tumor suppressor mRNA to restore its expression – have rarely been investigated in OS. Additionally, the potential competition between different ‘readers’ for the same m^6^A-modified transcript, which could lead to distinct functional outcomes, remains unaddressed.

Finally, the transformation of m^6^A research into clinical practice for OS is still in its infancy. Although preclinical research has confirmed the therapeutic potential of targeting m^6^A, there is an urgent need for clinical trials of m^6^A-targeted drugs in OS patients. Currently, several m^6^A-targeted inhibitors (e.g., FTO inhibitors like FB23-2, METTL3 inhibitors like STM2457) are under investigation in early-phase clinical trials for other cancers, but none have been tested in OS. Conducting clinical trials specifically for OS would aid in assessing the safety, pharmacokinetics, and effectiveness of these drugs in OS patients, and could also identify biomarkers to predict patient response, laying the foundation for personalized m^6^A-based OS therapies.

In conclusion, m^6^A modification is a central epigenetic hub regulating OS proliferation, metastasis, metabolism, PCD, and TME. Deciphering the exact mechanisms of m^6^A in OS will not only enhance our comprehension of OS pathogenesis but also expedite the creation of m^6^A-based diagnostic biomarkers and targeted treatments, ultimately improving the prognosis of OS patients.

## Data Availability

Data sharing is not applicable as no new data has been created in this study.
